# Methotrexate is not associated with increased liver cirrhosis in a population-based cohort of rheumatoid arthritis patients with chronic hepatitis B

**DOI:** 10.1038/srep22387

**Published:** 2016-03-01

**Authors:** Kuo-Tung Tang, Wei-Ting Hung, Yi-Hsing Chen, Ching-Heng Lin, Der-Yuan Chen

**Affiliations:** 1 Division of Allergy, Immunology and Rheumatology, Taichung Veterans General Hospital, Taichung, ROC; 2 Ph.D. Program in Translational Medicine, National Chung Hsing University, Taichung, ROC; 3 School of Medicine, National Yang-Ming University, Taipei, ROC; 4 Department of Medical Research, Taichung Veterans General Hospital, Taichung, ROC; 5 Institute of Microbiology and Immunology, Chung Shan Medical University, Taichung, ROC; 6 Institute of Biomedical Science, National Chung Hsing University, Taichung, ROC

## Abstract

A few studies showed that long-term methotrexate (MTX) use exacerbates liver fibrosis and even leads to liver cirrhosis in rheumatoid arthritis (RA) patients. We therefore conducted a population-based cohort study to investigate the impact of long-term MTX use on the risk of chronic hepatitis B (CHB)-related cirrhosis among RA patients. We analyzed data from the National Health Insurance Research Database in Taiwan and identified 631 incident cases of RA among CHB patients (358 MTX users and 273 MTX non-users) from January 1, 1998 to December 31, 2007. After a median follow-up of more than 6 years since the diagnosis of CHB, a total of 41 (6.5%) patients developed liver cirrhosis. We did not find an increased risk of liver cirrhosis among CHB patients with long-term MTX use for RA. Furthermore, there was no occurrence of liver cirrhosis among 56 MTX users with a cumulative dose ≧3 grams after 97 months’ treatment. In conclusion, our data showed that long-term MTX use is not associated with an increased risk for liver cirrhosis among RA patients with CHB. However, interpretation of the results should be cautious due to potential bias in the cohort.

Methotrexate (MTX) is a commonly used immunosuppressant in the treatment of many autoimmune diseases such as rheumatoid arthritis (RA), psoriasis and psoriatic arthritis^1^. Early studies have shown that the prevalence of liver cirrhosis was as high as 26% among MTX-treated psoriasis patients^2^, but later studies demonstrated a lower risk (between 0% and 10%)^3^,^4^,^5^. MTX has been regarded as the anchor drug in the treatment of RA^6^,^7^. Among RA patients, a few studies showed that long-term MTX use may exacerbate liver fibrosis in sequential liver biopsies among RA patients^8^,^9^, whereas other studies did not find such an association^10^,^11^,^12^,^13^,^14^. Nonetheless, the risk of MTX-related liver cirrhosis in RA patients seems to be lower than that in psoriasis patients^15^. A meta-analysis demonstrated that 2.7% of RA patients would develop severe fibrosis or cirrhosis after 55 months’ MTX treatment^16^. Based on these findings, the American College of Rheumatology (ACR) does not suggest routine performance of liver biopsy in RA patients who use MTX^17^.

Alcohol consumption, diabetes, obesity, chronic viral hepatitis and hepatotoxic medications have been reported to be etiological factors of liver cirrhosis^18^. In Taiwan, where hepatitis B is endemic^19^, the impact of long-term MTX use on the risk of chronic hepatitis B (CHB)-related cirrhosis among RA patients has not been investigated. We hypothesized that long-term MTX use increases the risk for liver cirrhosis among RA patients with CHB. We therefore conducted a retrospective cohort study using data from a nationwide health insurance database.

## Methods

### Patients

This retrospective cohort study analyzed data obtained from the National Health Insurance Research Database (NHIRD)^20^,^21^, established by the Taiwan National Health Research Institute. The NHIRD contains comprehensive health care data from more than 99% of the entire Taiwanese population of 24 million. Diseases in the NHIRD are coded with the International Classifications of Diseases, Ninth Revision, Clinical Modification (ICD-9-CM) codes. In addition, we ensured that RA diagnosis was made according to the 1987 ACR criteria^22^ using the Catastrophic Illness Patient Database (CIPD), a registry including RA certified by two rheumatologists. Since the NHIRD contains only de-identified secondary data, the need for informed consent from individuals was waived.

**Figure 1:**
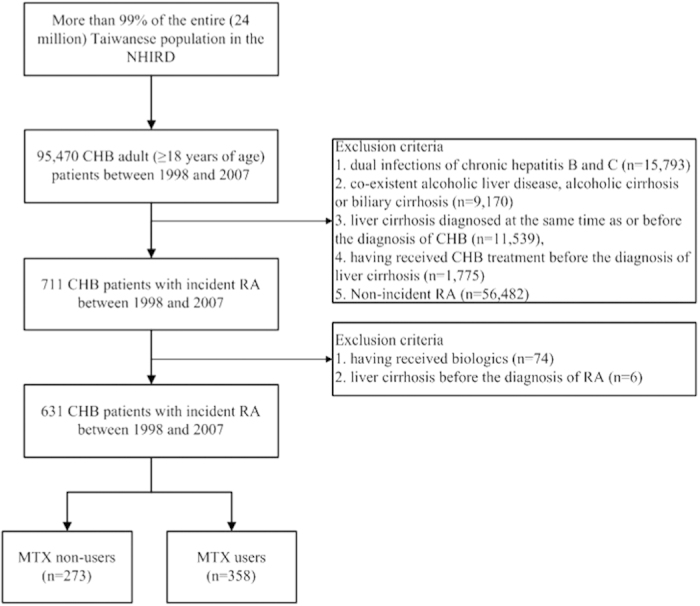
The flow chart of identification of RA patients with CHB. CHB, chronic hepatitis B; MTX, methotrexate; NHIRD, the National Health Insurance Research Database; RA, rheumatoid arthritis.

The study flowchart is illustrated in Fig. 1. We identified 95,470 adult patients who were diagnosed with CHB (ICD-9-CM codes 070.2, 070.3 or V02.61) in Taiwan from January 1, 1998 to December 31, 2007. Liver cirrhosis was defined as ICD-9-CM code 571.5. Exclusion criteria included dual infections of CHB and chronic hepatitis C (ICD-CM codes 070.54, 070.70 or V02.62), co-existent alcoholic liver disease (ICD-9-CM codes 571.0, 571.1 or 571.3), alcoholic cirrhosis (ICD-9-CM code 571.2) or biliary cirrhosis (ICD-9-CM code 571.6), pre-existing liver cirrhosis at the time of or before the diagnosis of CHB, and CHB treatment (lamivudine, adefovir dipivoxil, entecavir, telbivudine, tenofovir, or peginterferon alfa-2a) before the diagnosis of liver cirrhosis. We then identified 711 incident cases of RA among these CHB patients. Patients with liver cirrhosis before the diagnosis of RA or with prescription of biologics (etanercept, adalimumab, golimumab, abatacept, tocilizumab or rituximab) before the diagnosis of liver cirrhosis were excluded. In total, 631 CHB patients with incident RA (consisting of 358 MTX users and 273 MTX non-users) were identified and included in the final analysis. This study was conducted in compliance with the Declaration of Helsinki and has been approved by the Institutional Review Board of Taichung Veterans General Hospital (IRB TCVGH No. CE15124B).

### Clinical parameters

Risk factors for liver cirrhosis, such as non-alcoholic fatty liver disease (NAFLD, ICD-9-CM code 571.8), diabetes mellitus (ICD-9-CM code 250) and dyslipidemia (ICD-9-CM code 272), as well as comorbidities such as hypertension (ICD-9-CM codes 401–405) were documented for each patient. Diagnosis of decompensated liver cirrhosis in all cases was confirmed by inclusion in the CIPD. One of the following criteria is needed for patients with liver cirrhosis to be registered in the CIPD: (1) intractable ascites, (2) variceal bleeding, or (3) hepatic coma or liver decompensation.

### Statistics

Statistical analysis was conducted using SAS software version 9.3 (SAS Institute, Cary, NC, USA.). All quantitative data are presented as means plus the standard deviation unless otherwise specified. For numerical variables, comparisons between subgroups of patients were performed using Student’s t test. For categorical variables, comparisons between subgroups of patients were performed using Chi-squared test. Hazard ratios of developing liver cirrhosis since the diagnosis of CHB between subgroups of RA patients with CHB were calculated using the Cox proportional hazards model after adjustment for age, gender and comorbidities such as NAFLD, diabetes mellitus, dyslipidemia and hypertension. Kaplan-Meier survival analysis and the log rank test were used to compare the probability of liver cirrhosis-free survival since the diagnosis of CHB between MTX non-users and MTX users with different cumulative doses. A two-tailed p value less than 0.05 was considered statistically significant.

## Results

### Baseline characteristics of RA patients with CHB

Table 1 illustrates the baseline characteristics of RA patients with CHB. Leflunomide, sulfasalazine and corticosteroids were prescribed more often in MTX users than in MTX non-users, implying more severe disease activity of RA among MTX users. In our 358 MTX users, the average cumulative MTX dosage was 1.5 ± 1.6 grams after a mean of 40 months. We categorized them into 3 groups based on the cumulative MTX dose. The mean durations of treatment were 16, 55 and 97 months and the mean weekly doses were 8.5, 9.9 and 11.7 mg, within these groups of MTX users (MTX cumulative dose <1.5 grams, 1.5–3.0 grams and 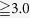 grams). After a median follow-up of more than 6 years since the diagnosis of CHB, a total of 41 (6.5%) patients developed liver cirrhosis: 22 (6.1%) of 358 MTX users and 19 (7.0%) of 273 MTX non-users. Among the 22 MTX users who developed liver cirrhosis, 18 patients had a cumulative dose of ≦1.5 grams and 4 patients had a cumulative dose of ≧1.5 grams and <3.0 grams. Four (1.1%) of 358 MTX users and 2 (0.7%) of 273 MTX non-users developed decompensated liver cirrhosis. Notably, there was no occurrence of liver cirrhosis among 56 MTX users with a cumulative dose of ≧3 grams.

### Multivariate analysis of risk factors for liver cirrhosis among RA patients with CHB

Table 2 illustrates risk factors for liver cirrhosis in RA patients with CHB utilizing the Cox proportional hazards model. Older age at diagnosis of CHB and male sex were risk factors for liver cirrhosis. There was a trend toward an increased hazard for liver cirrhosis in patients with NAFLD, with an hazard ratio of 9.21 (95% confidence interval [CI]: 1.18–71.66). The concomitant use of MTX was not associated with an increased hazard of liver cirrhosis (hazard ratio: 0.90, 95% CI: 0.48–1.68), even for MTX users with a cumulative dose of ≧1.5 grams (hazard ratio: 0.39, 95% CI: 0.13–1.17, Appendix 1), when compared to MTX non-users.

### Subgroup analysis of the incidence rates of liver cirrhosis among RA patients with CHB

Table 3 illustrates the subgroup analysis of the incidence rates of liver cirrhosis among RA patients with CHB. Among patients with diabetes mellitus, there was a lower incidence rate of liver cirrhosis among MTX users compared to MTX non-users (8.1 versus 26.0 events per 1000 person-years, p = 0.041). Among patients with concomitant use of sulfasalazine, we observed a trend toward a higher incidence rate of liver cirrhosis among MTX users when compared to MTX non-users (11.5 versus 4.8 events per 1000 person-years, p = 0.083).

### The probability of liver cirrhosis-free survival among RA patients with CHB

**Figure 2:**
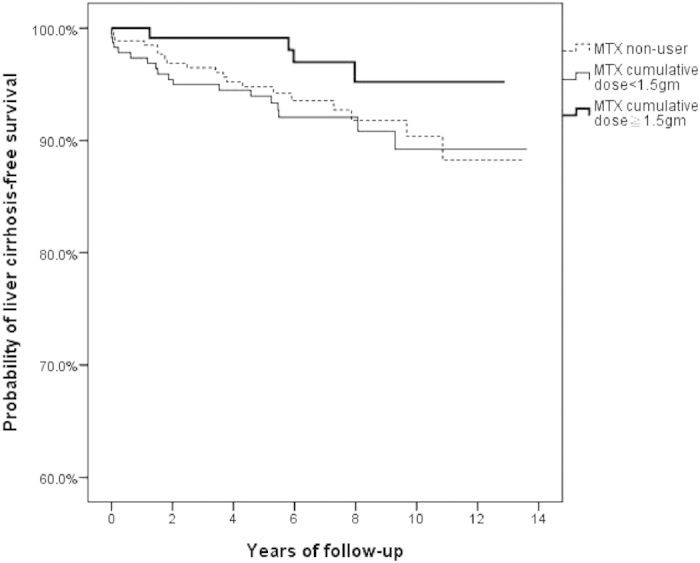
Probability of liver cirrhosis-free survival among MTX users with different cumulative doses and MTX non-users (p = 0.176, log-rank test). MTX, methotrexate.

Figure 2 shows the Kaplan-Meier survival analysis of liver cirrhosis-free survival with respect to MTX use among RA patients with CHB. There was no statistically significant difference between MTX non-users and MTX users with different cumulative doses (p = 0.176, log-rank test), although there was a trend toward a higher probability of liver cirrhosis-free survival among MTX users with a cumulative dose of ≧1.5 grams when compared to MTX non-users and MTX users with a cumulative dose of <1.5 grams.

## Discussion

This population-based cohort study investigated the impact of long-term MTX use on the risk of liver cirrhosis among RA patients with CHB. Our results demonstrate no significant increase of liver cirrhosis in RA patients with CHB who received long-term MTX treatment. However, no firm conclusion can be drawn due to potential bias in the cohort.

Among RA patients with CHB in Taiwan, 358 (57%) of 631 patients (excluding those patients who had received biologics) had received MTX to treat RA. The proportion of MTX used in our study was lower than that reported in a multi-national cross-sectional study of RA patients^23^, perhaps due to the reluctance of physicians to use MTX in these patients. However, MTX was still used by over half of RA patients with CHB, reflecting its fundamental role in RA treatment even in an endemic area for CHB. As for comorbidities, the prevalence of NAFLD in our RA patients was lower than rates reported in earlier studies that described liver biopsy findings in RA patients^24^,^25^, probably due to suboptimal monitoring of RA patients for comorbidities^23^.

After a median follow-up of more than 6 years since the diagnosis of CHB, 41 (6.5%) of 703 RA patients with CHB developed liver cirrhosis. The incidence of liver cirrhosis in our study was higher than that in the general population with CHB (3.0% after a mean follow-up of more than 9 years)^26^. However, it should be noted that RA patients with CHB were predominantly female and older in age. The discrepancy in demographic characteristics and the lack of adjustment for confounding variables should be taken into consideration when viewing our higher incidence of liver cirrhosis in CHB patients with comorbid RA. Further epidemiological study is needed.

Previous studies indicated that the risk for liver cirrhosis after long-term MTX therapy is low among RA patients^16^. But it was unclear whether there was an increased risk among patients with comorbidities that contribute to liver cirrhosis, such as CHB. In our study, a comparable proportion of MTX users and MTX non-users developed liver cirrhosis (6.2% vs. 7.0%) and decompensated liver cirrhosis (1.1% vs. 0.7%). In the Cox proportional hazards model, older age and male sex were identified as risk factors of developing liver cirrhosis among RA patients with CHB, which is compatible with previous findings^27^. However, we did not find an increased hazard for liver cirrhosis or a significant difference in liver cirrhosis-free survival among MTX users when compared to MTX non-users, although higher proportions of concomitant use of hepatotoxic drugs such as sulfasalazine and leflunomide^1^, and corticosteroids, which are associated with hepatitis B reactivation^28^, were found in MTX users. Even for those patients who used a higher cumulative dose of MTX (≧1.5 grams), there was no such increased risk of liver cirrhosis. Furthermore, there was no occurrence of liver cirrhosis among 56 MTX users with a cumulative dose of ≧3 grams after 97 months’ treatment. Taken together, these findings implied that long-term MTX use was not associated with an increased risk of liver cirrhosis among RA patients with CHB. Our results are consistent with previous observational studies demonstrating the low risk of MTX-related liver cirrhosis in RA patients^16^.

However, it is difficult to draw a firm conclusion from our data since such cohort is easily biased despite efforts to adjust for confounding variables. Nevertheless, analysis using a real-world database can still provide insight regarding the issue. We gave a possible explanation for our observations^16^. Compared to MTX users, MTX non-users seemed to have less severe disease activity, which is associated with higher body mass index^29^,^30^. This was correlated with the development of NAFLD^31^, which contributed to the development of liver cirrhosis^31^. The potential impact of NAFLD in our study is reflected in the increased hazard of liver cirrhosis among MTX non-users when compared to MTX users in the subgroup of patients with diabetes mellitus, which is associated with NAFLD^31^. Although we did not find a difference in the prevalence of diagnosed NAFLD between MTX users and non-users, the true prevalence of NAFLD might be underestimated due to the reason described above. Interestingly, a recent analysis of individuals who had been listed for, and/or received liver transplantation in the US., found that the burden of end-stage MTX-related liver disease is exceedingly small and the risk factor profile of MTX-related liver disease is similar to that of non-alcoholic steatohepatitis (NASH), suggesting a common pathogenesis^32^.

Our study has a number of advantages. First, it is difficult to conduct a randomized control trial to evaluate the impact of long-term MTX use on the risk of CHB-related liver cirrhosis. Therefore our data, based on a population-based cohort from a database containing the healthcare records of 24 million people, can still provide important insight. Second, unlike previous studies that mainly focused on liver fibrosis^8^,^9^,^10^,^11^,^12^,^13^,^14^, our study investigated clinically manifest liver cirrhosis, which leads to morbidities and mortality^18^.

Our study has some limitations. First, a longer follow-up period with a higher cumulative MTX dose may be required to observe the contribution of MTX to the risk of HBV-related liver cirrhosis. Therefore, a study with a longer follow-up is needed to confirm our results. Second, previous studies demonstrated that the adherence rate to MTX ranged from 59% (underuse) to 107% (overuse)^33^. However, it is difficult to evaluate the adherence to medications in an observational study, particularly in a claims database. Therefore the medication effect might be underestimated due to nonadherence. Third, anthropometric measurements are not available in the NHIRD and therefore we could not evaluate the impact of body mass index, which is associated with the development of NAFLD^31^. Fourth, daily alcohol consumption is not documented in the NHIRD. However, the prevalence of alcohol-related health problems seems to be lower in Taiwan compared with Western countries^34^. In addition, patients with co-existent alcoholic liver disease and/or alcoholic cirrhosis were excluded from the study cohort. Therefore, the lack of documented daily alcohol consumption was not expected to markedly bias our study results. Fifth, it is possible that some patients with active CHB presented with elevated liver enzymes^27^, thereby preventing MTX use and contributing to a higher risk for liver cirrhosis among MTX non-users^27^. However, patients with active CHB (receiving CHB treatment) were excluded from this cohort and such selection bias was reduced.

In conclusion, our data showed that long-term MTX use is not associated with an increased risk for liver cirrhosis among RA patients with CHB. However, interpretation of the results should be cautious due to potential bias in the cohort.

## Additional Information

**How to cite this article**: Tang, K.-T. *et al.* Methotrexate is not associated with increased liver cirrhosis in a population-based cohort of rheumatoid arthritis patients with chronic hepatitis B. *Sci. Rep.* **6**, 22387; doi: 10.1038/srep22387 (2016).

## Supplementary Material

Supplementary Information

## Figures and Tables

**Table 1:** Baseline characteristics of rheumatoid arthritis patients with chronic hepatitis B.

Variables	MTX users (n = 358)	MTX non-users (n = 273)
Age (years)	53.3 ± 12.7	54.8 ± 13.7#
Gender		
Female	274 (77%)	206 (75%)
Male	84 (23%)	67 (25%)
Comorbidity		
NAFLD	2 (1%)	3 (1%)
Diabetes mellitus	58 (16%)	37 (14%)
Dyslipidemia	55 (15%)	46 (17%)
Hypertension	95 (27%)	79 (29%)
Concomitant medications		
Leflunomide	39 (11%)	4 (2%)*
Azathioprine	10 (3%)	12 (4%)
Sulfasalazine	190 (53%)	91 (33%)*
Corticosteroids	263 (74%)	107 (39%)*
MTX		
Cumulative dose <1.5 grams	235 (66%)	NA
Cumulative dose 1.5–3.0 grams	67 (19%)	NA
Cumulative dose ≧3.0 grams	56 (16%)	NA
Liver cirrhosis	22 (6%)	19 (7%)
Decompensated liver cirrhosis	4 (1%)	2 (1%)
Median follow-up period (years)	6.8	6.2##

MTX: methotrexate; NA: not avalaible; NAFLD: non-alcoholic fatty liver disease

^*^p < 0.0001.

^#^at diagnosis of chronic hepatitis B.

^##^since the diagnosis of chronic hepatitis B.

**Table 2:** Multivariate analysis for liver cirrhosis in rheumatoid arthritis patients with chronic hepatitis B.

Variables	Adjusted HR (95% CI)
Age at diagnosis of chronic hepatitis B (years)	1.07 (1.04–1.11)
Gender	
Female	1.00
Male	3.90 (2.08–7.29)**
Concomitant MTX use	0.90 (0.48–1.68)
Comorbidity	
NAFLD	9.21 (1.18–71.66)*
Diabetes mellitus	1.32 (0.56–3.08)
Dyslipidemia	0.42 (0.15–1.17)
Hypertension	1.28 (0.63–2.58)

*p < 0.05; **p < 0.001.

CI: confidence interval; MTX: methotrexate; NAFLD:

non-alcoholic fatty liver disease.

**Table 3:** Subgroup analysis for new-onset liver cirrhosis in rheumatoid arthritis patients with chronic hepatitis B.

Variables	MTX users	MTX non-users	Adjusted hazard ratio (95% CI)*
	Incidence rate of liver cirrhosis (per 1,000 person-years)	Incidence rate of liver cirrhosis (per 1,000 person-years)	
All patients	9.1	10.5	0.92 (0.48–1.77)
Age at the diagnosis of chronic hepatitis B (years)			
18–44	1.5	0.0	NA
45–64	5.8	13.7	0.48 (0.19–1.25)
≧65	35.0	20.6	1.41 (0.50–4.01)
Gender			
Female	5.7	6.4	1.16 (0.46–2.93)
Male	23.0	25.2	0.74 (0.29–1.86)
Comorbidity			
Diabetes mellitus	8.1	26.0	0.16 (0.03–0.92)
Dyslipidemia	2.5	14.8	0.17 (0.02–1.76)
Hypertension	12.9	18.0	0.77 (0.28–2.10)
Concomitant medications			
Sulfasalazine	11.5	4.8	3.25 (0.86–12.27)
Corticosteroids	3.9	24.9	1.41 (0.58–3.43)

CI: confidence interval; MTX: methotrexate; NA: not available.

^*^adjusted for age, gender, diabetes mellitus, dyslipidemia, hypertension and medications such as sulfasalazine and corticosteroids.
