# Supplementary material for: Methotrexate is not associated with increased liver cirrhosis in a population-based cohort of rheumatoid arthritis patients with chronic hepatitis B
Source: Sci Rep. 2016 Mar 1;6:22387. doi: 10.1038/srep22387 (PMC4772158; doi:10.1038/srep22387)
Supplement: Supplementary Information [file srep22387-s1.doc]

**Title: Methotrexate is not associated with increased liver cirrhosis in a population-based cohort of rheumatoid arthritis patients with chronic hepatitis B.**

Kuo-Tung Tang^1,2^, Wei-Ting Hung^1,3^, Yi-Hsing Chen^1,3^, [Ching-Heng Lin](http://rheumatology.oxfordjournals.org/search?author1=Ching-Heng+Lin&sortspec=date&submit=Submit)^4,*^, Der-Yuan Chen^1,3,5,6,*^

1. Division of Allergy, Immunology and Rheumatology, Taichung Veterans General Hospital, Taichung, R.O.C.
2. Ph.D. Program in Translational Medicine, National Chung Hsing University, Taichung, R.O.C.
3. School of Medicine, National Yang-Ming University, Taipei, R.O.C.
4. Department of Medical Research, Taichung Veterans General Hospital, Taichung, R.O.C.
5. Institute of Microbiology and Immunology, Chung Shan Medical University, Taichung, R.O.C.
6. Institute of Biomedical Science, National Chung Hsing University, Taichung, R.O.C

^*^Correspondence and reprint requests to:

Prof. Der-Yuan Chen, Division of Allergy, Immunology and Rheumatology, Taichung Veterans General Hospital, No. 1650, Sec. 4, Taiwan Blvd., Taichung 40705, R.O.C.

TEL: 886-4-23592525 ext. 4000

FAX: 886-4-23503285

E-mail: [dychen@vghtc.gov.tw](mailto:dychen@vghtc.gov.tw)

Co-correspondence

Prof. [Ching-Heng Lin](http://rheumatology.oxfordjournals.org/search?author1=Ching-Heng+Lin&sortspec=date&submit=Submit), Department of Medical Research, Taichung Veterans General Hospital, No. 1650, Sec. 4, Taiwan Blvd., Taichung 40705, R.O.C.

TEL: 886-4-23592525 ext. 4089

FAX: 886-4-23592705

E-mail: epid@vghtc.gov.tw

**Appendix 1.** Incorporating subgroups of MTX users with different

cumulative doses in the multivariate analysis for liver cirrhosis in

rheumatoid arthritis patients with chronic hepatitis B.

| Variables | Adjusted HR (95% CI) |
| --- | --- |
| Age at diagnosis of chronic hepatitis B (years) | 1.07 (1.04-1.11)** |
| Gender |  |
| Female | 1.00 |
| Male | 4.44 (2.36-8.39)** |
| MTX nonusers | 1.00 |
| MTX users |  |
| MTX cumulative dose < 1.5 grams | 1.26 (0.65-2.45) |
| MTX cumulative dose ≧ 1.5 grams | 0.39 (0.13-1.17) |
| Comorbidity |  |
| NAFLD | 8.37 (1.08-65.04)* |
| Diabetes mellitus | 1.18 (0.51-2.73) |
| Dyslipidemia | 0.47 (0.17-1.27) |
| Hypertension | 1.22 (0.61-2.45) |

*p<0.05; **p<0.001

CI: confidence interval; MTX: methotrexate; NAFLD:

non-alcoholic fatty liver disease
